# Supplementary material for: Optogenetic control of small GTPases reveals RhoA mediates intracellular calcium signaling
Source: J Biol Chem. 2021 Jan 13;296:100290. doi: 10.1016/j.jbc.2021.100290 (PMC7949103; doi:10.1016/j.jbc.2021.100290)

Supporting information for “**Optogenetic control of Small GTPases reveals RhoA mediates intracellular calcium signaling**” by H. Inaba, Q. Miao and T. Nakata.

Supplementary Figure Legends

Figure S1. Time courses of mCherry-SspB-LARG-DH translocation in response to irradiation with different wavelengths and laser power. HeLa cells expressing the mCherry version of opto-RhoA were observed via confocal microscopy in Leibovitz’s L-15 medium. Cells were irradiated by a 458- (**A, D**), 488- (**B**), or 515-nm (**C**) laser at the indicated power every 10 s over the period of 60–170 s. Cytosolic mCherry-SspB-LARG-DH levels were quantified, and the fluorescence intensity was normalized by the intensity at 50 s. Data are presented as the mean ± SD. The same cells were observed in each panel. N = 5, 4, 5, and 5 cells, respectively.

Figure S2. Time course of opto-GTPase translocation. HeLa cells expressing the mCherry version of opto-GTPases were observed (Figure S1). Data are presented as the mean ± SD. N = 6, 5, 6, 7, 3, and 5 cells, respectively. A scale-modified and merged graph, including opto-RhoA (Figure S1), is presented to compare the kinetics of opto-GTPases (**G**).

Figure S3. Time courses of GTPase activity upon opto-GTPase activation. Supplementary Figure for Figure 2. Time courses of RhoA (**A**), Rac1 (**B**), Cdc42 (**C**), HRas (**D**), Rap1A (**E**), and RalB (**F**) activity for all opto-GTPases were merged into graphs. Time courses for opto-control and opto-GTPases corresponding to GTPase biosensors are the same data presented in Figure 2. Data are presented as the

mean ± SD.

Figure S4. Opto-Ras induced ERK translocation to the nucleus. HeLa cells transiently expressing opto-GTPases and ERK2-mCherry were serum starved for 6–9 h and observed using a confocal microscope in Leibovitz's L-15 medium. Opto-GTPases were activated with the help of multi-argon 458-nm laser irradiation every 10 s during a period of 60–350 s. Time course of the relative fluorescence intensity of nuclear ERK2-mCherry (**A**). Changes of the fluorescence intensity of nuclear ERK2-mCherry after 5 min of blue light-irradiation (**B**). Data presented as individual points and the mean ± SD. N = 19, 20, 13, 18, and 11 cells, respectively. Data were analyzed using one-way ANOVA followed by Dunnett's test between opto-control and other groups. ANOVA $F = 18.32$ and $p < 0.0001$. ***,, $p < 0.001$; ns, not significant; Dunett's test.

Figure S5. Intracellular calcium changes in HeLa, MDCK, and HEK293T cells during RhoA activation. Representative images of HeLa, MDCK, and HEK293T cells expressing opto-RhoA and R-GECO1 in RPE1 cells. From time 0 s, opto-RhoA was activated by a multi-argon 458-nm laser every 10 s. Scale bar, 50 µm.

Figure S6. Expression of msPLCε-ECFP rescued opto-RhoA-induced calcium transients in PLCε-depleted cells. siRNA-mediated PLCε-depleted RPE1 and HeLa cells transiently expressing ECFP or msPLCε-ECFP and R-GECO1 were observed (see Figure 3). Data are presented as means ± SD from three independent experiments. In total, >200 cells were analyzed for each condition. **, $p < 0.01$; *, $p < 0.05$; two-tailed unpaired Student's t-test.

Supplementary Movie Legends

Movie 1. Opto-RhoA can control RhoA activity both in time and space. Supplemental movie for Figure 1C.

Movie 2. Opto-RhoA induces the calcium transients in various cell types. Supplemental movie for Figure 3 and S5.

Movie 3. Opto-5-ptase activation decrease membrane PI(4,5)P_2. Supplemental movie for Figure 6E.

Movie 4. Opto-RhoA activation marginally affects membrane PI(4,5)P_2. Supplemental movie for Figure 6E.

Movie 5. Opto-RhoA-mediated calcium transients translocate NFAT from cytoplasm to the nucleus. Supplemental movie for Figure 7A.

Inaba et al., Figure S1

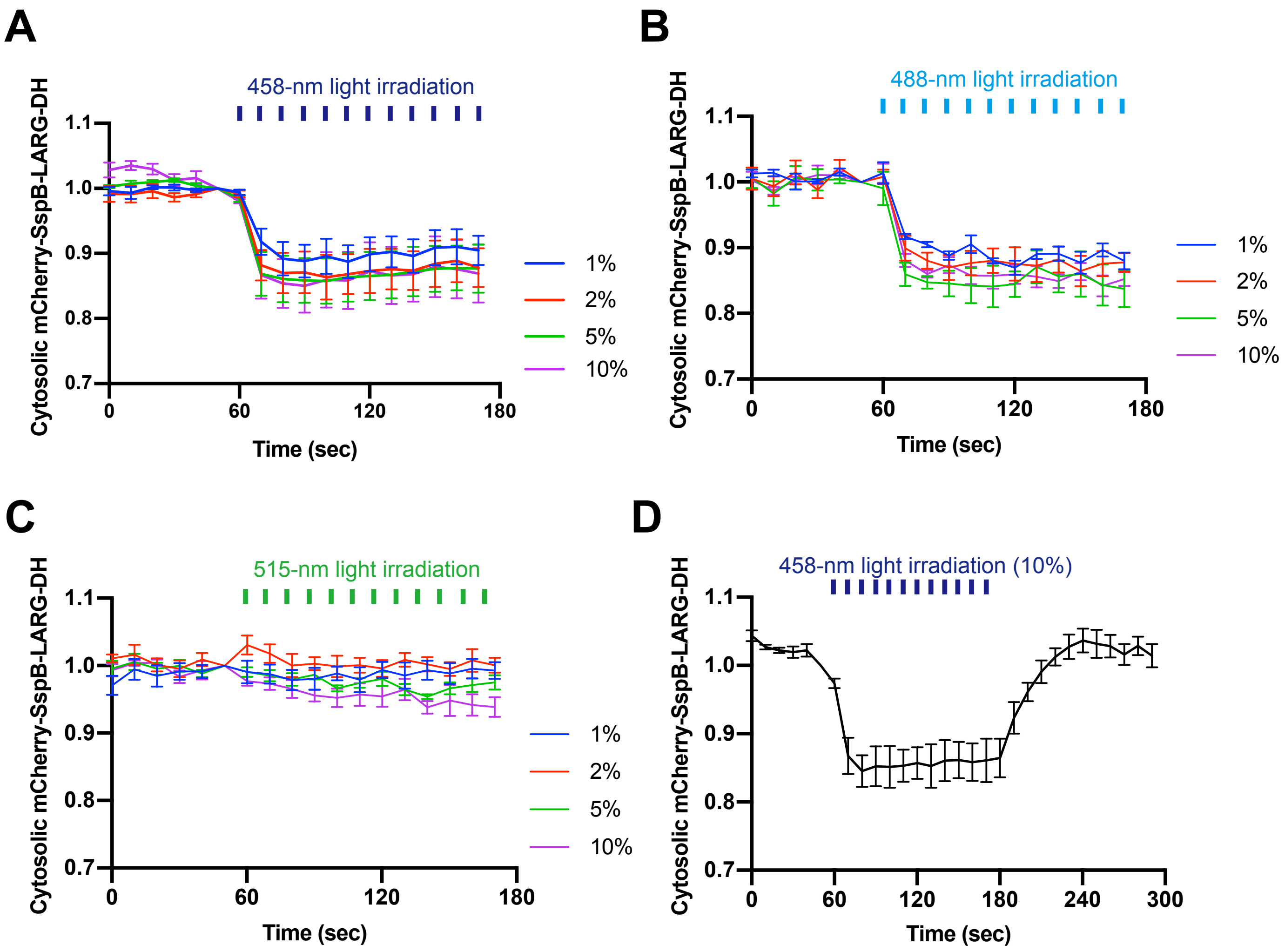

Inaba et al., Figure S2

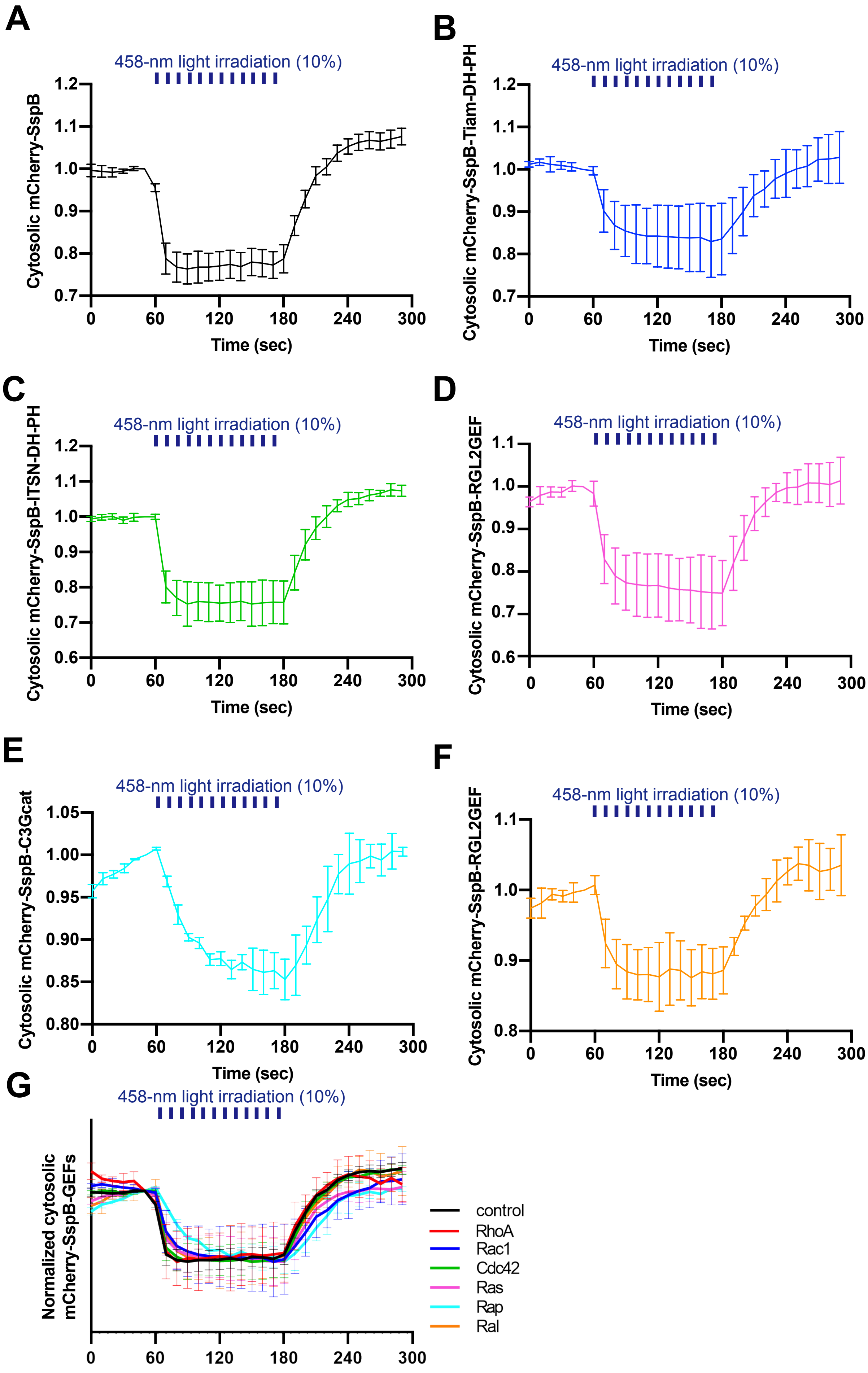

Inaba et al., Figure S3

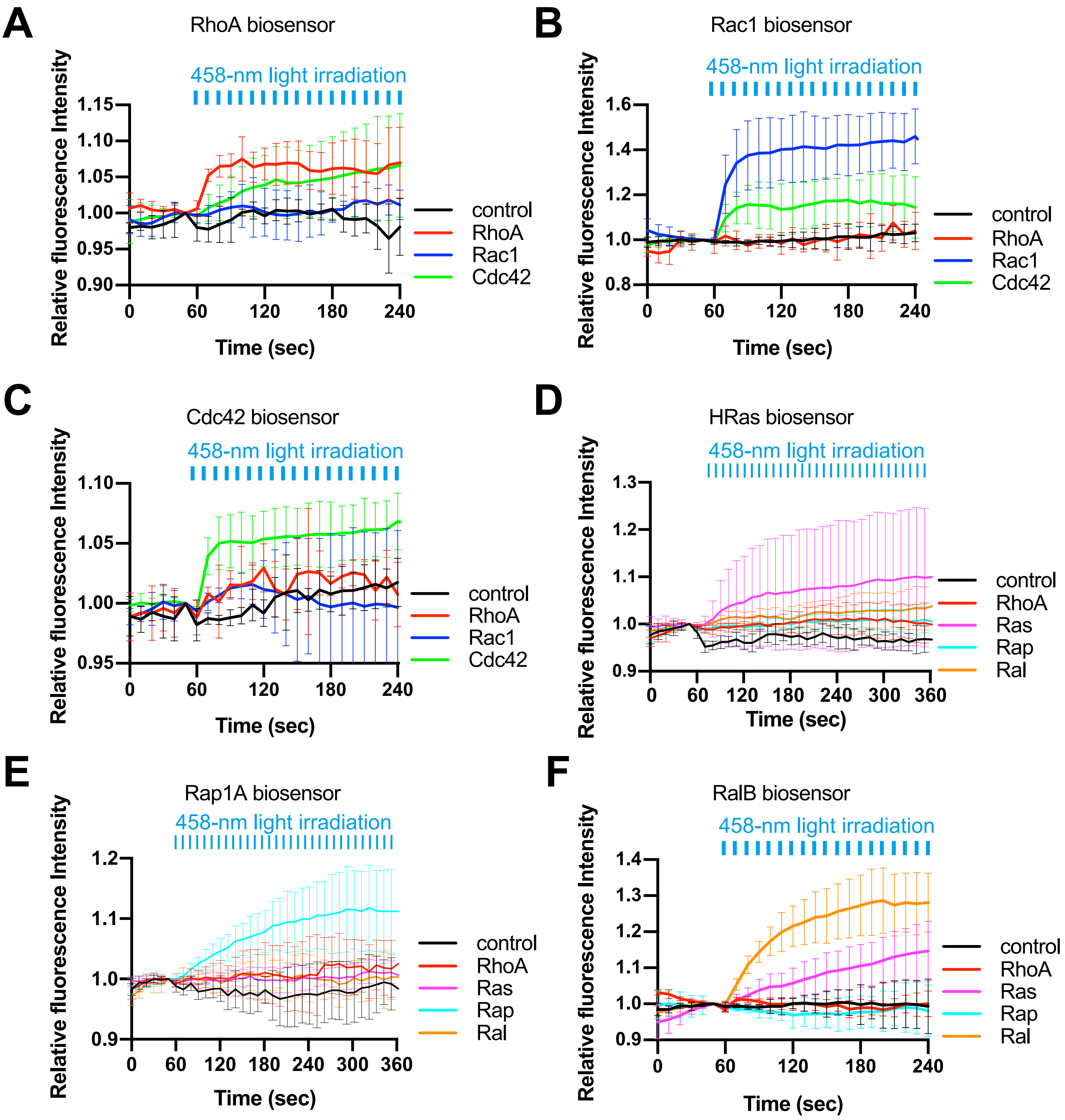

Inaba et al., Figure S4

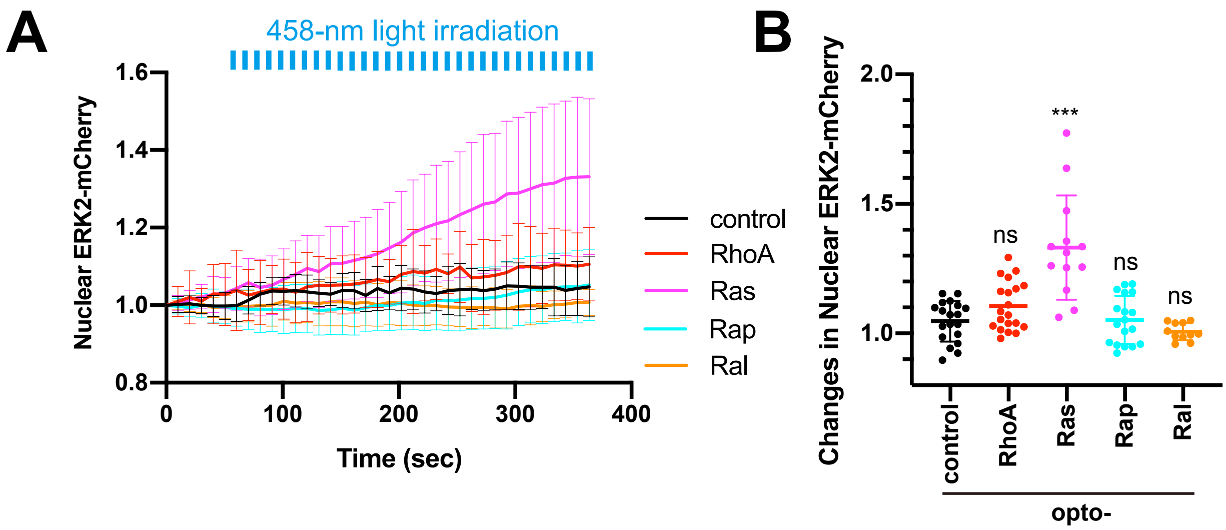

Inaba et al., Figure S5

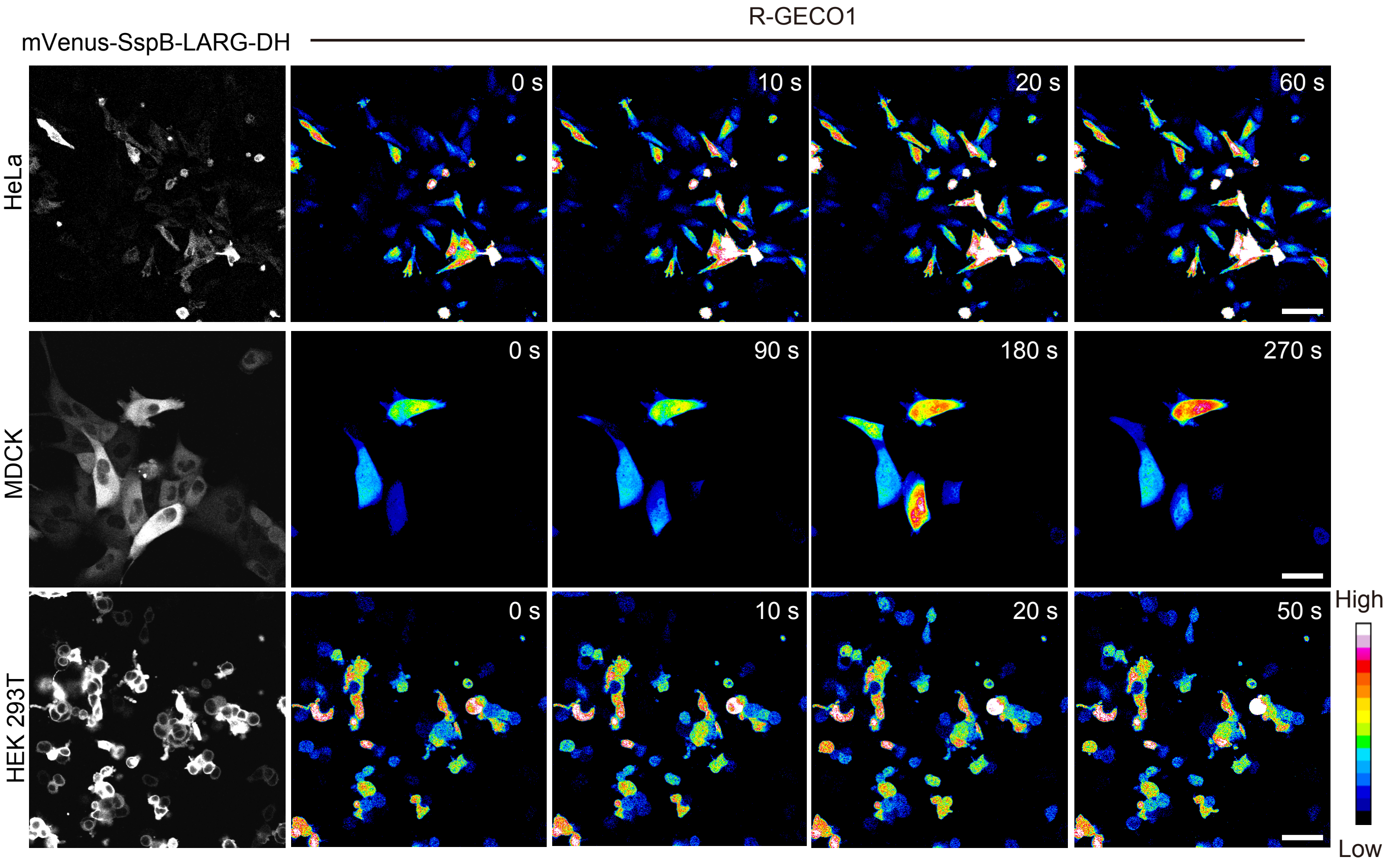

Inaba et al., Figure S6

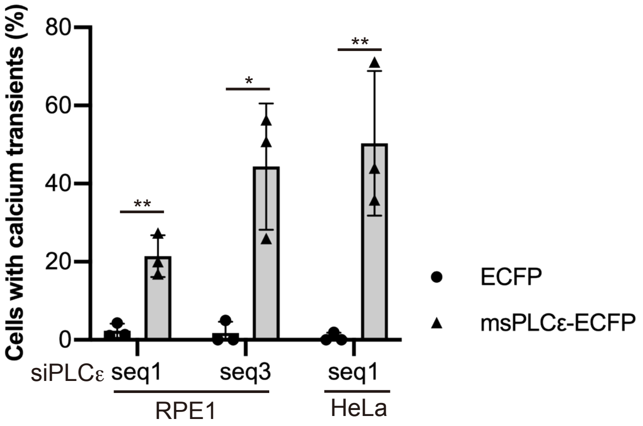

Supplement: Figures S1–S6 [file mmc4.pdf]
